# Supplementary material for: A time-resolved multi-omics atlas of transcriptional regulation in response to high-altitude hypoxia across whole-body tissues
Source: Nat Commun. 2024 May 10;15:3970. doi: 10.1038/s41467-024-48261-w (PMC11087590; doi:10.1038/s41467-024-48261-w)
Supplement: Supplementary file 1 — Supplementary Information [file 41467_2024_48261_MOESM1_ESM.pdf]

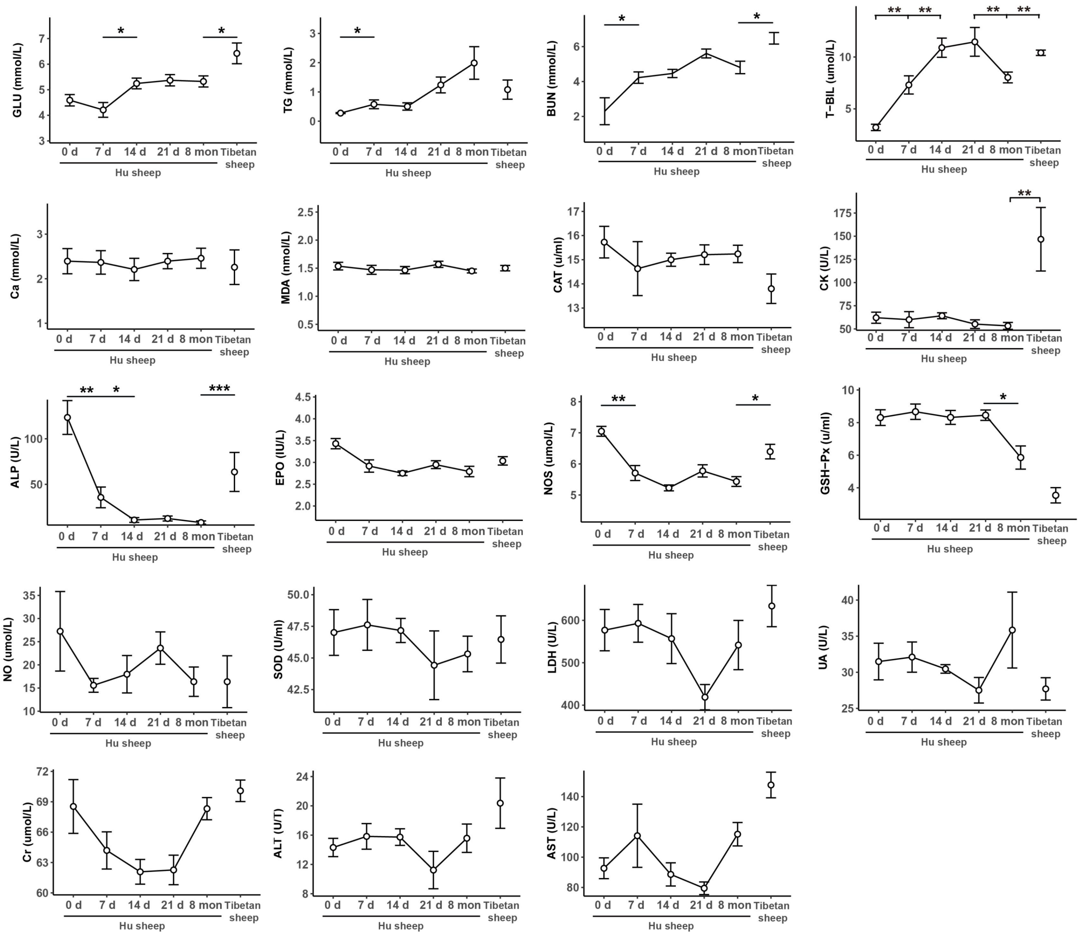

Supplementary Fig. 1 The changes of phenotypes. The change in value of bio-indicators with time. GLU, glucose; TG, triglycerides; ALT, alanine transaminase; AST, aspartate aminotransferase; T-BIL, total bilirubin; ALP, alkaline phosphatase; LDH, lactate dehydrogenase; UA, uric acid; BUN, blood urea nitrogen; Cr, creatinine; Ca, calcium; SOD, superoxide dismutase; GSH-Px, glutathione peroxidase; CAT, catalase; NO, nitric oxide; MAD, malondialdehyde; EPO, erythropoietin. Data are represented as mean values ± standard error (n = 10). P values from two-sided Wilcoxon rank sum test, * $P < 0.05$, ** $P < 0.001$, *** $P < 0.0001$. Source Data are provided as Source Data file.

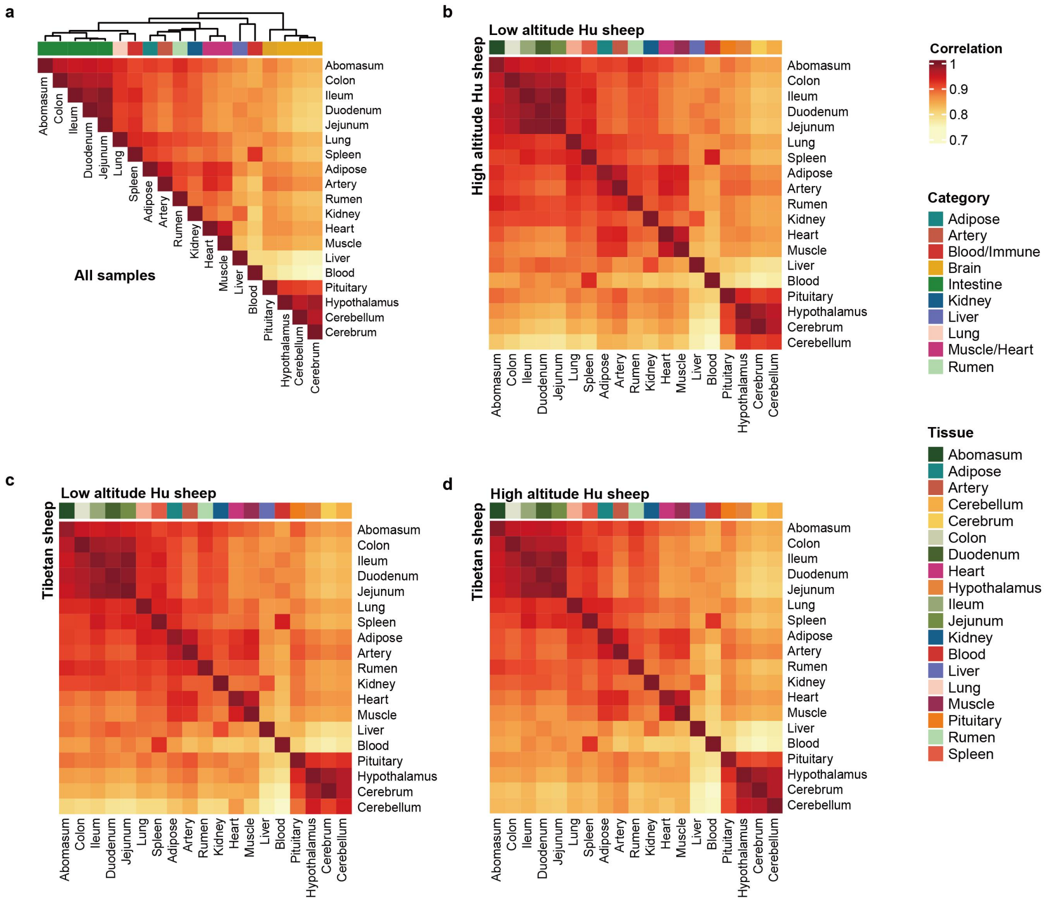

Supplementary Fig. 2 Hierarchical clustering of RNA-Seq samples. (a) Hierarchical clustering of tissues (n = 1,277) based on Pearson's correlation of median value of expression. (b-d) Similar with a, hierarchical clustering of tissues based on Pearson's correlation of median value of expression between low altitude Hu sheep and high altitude Hu sheep (b), between low altitude Hu sheep and Tibetan sheep (c) and between high altitude Hu sheep and Tibetan sheep (d). Source Data are provided as Source Data file.

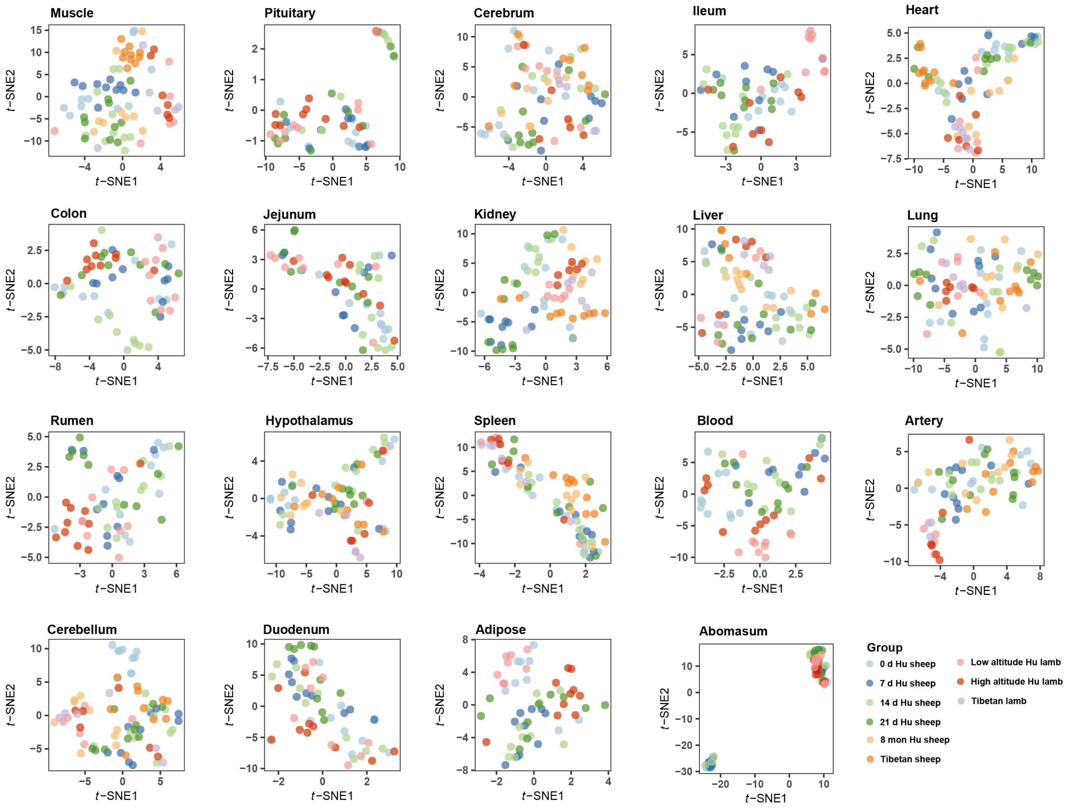

Supplementary Fig. 3 Sample clustering within each tissue. *t*-Distributed Stochastic Neighbor Embedding (*t*-SNE) clustering between groups within each tissue.

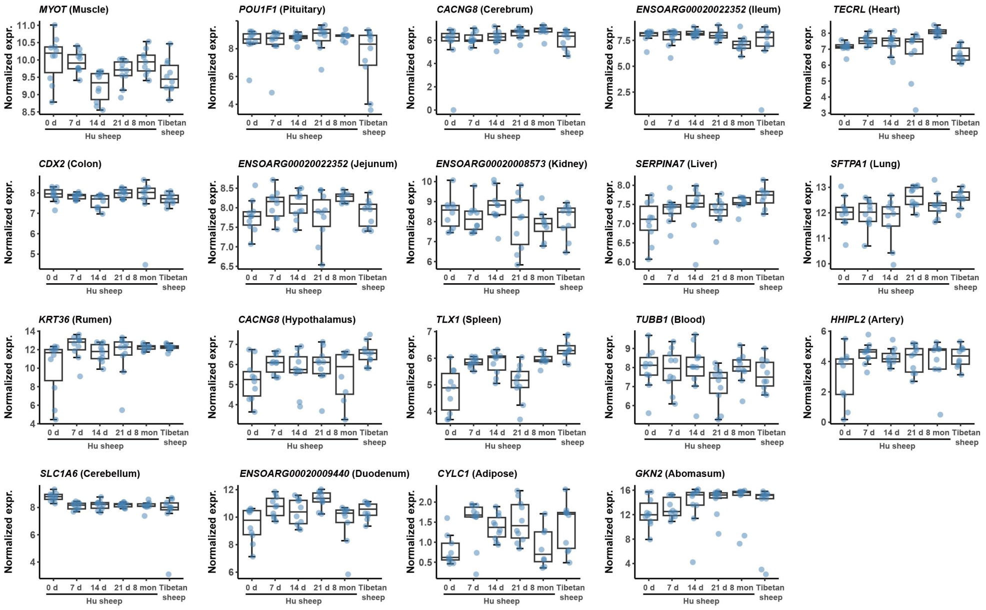

Supplementary Fig. 4 Example of tissue-specific expressed gene. The expression level of tissue-specific gene with time in corresponding tissue. Boxplots are represented by minima, 25% quantile, median, 75% quantile, and maxima. Each blue dot represents the expression of a sample in each group (n = 10). Gene with top t-statistic is shown for each tissue.

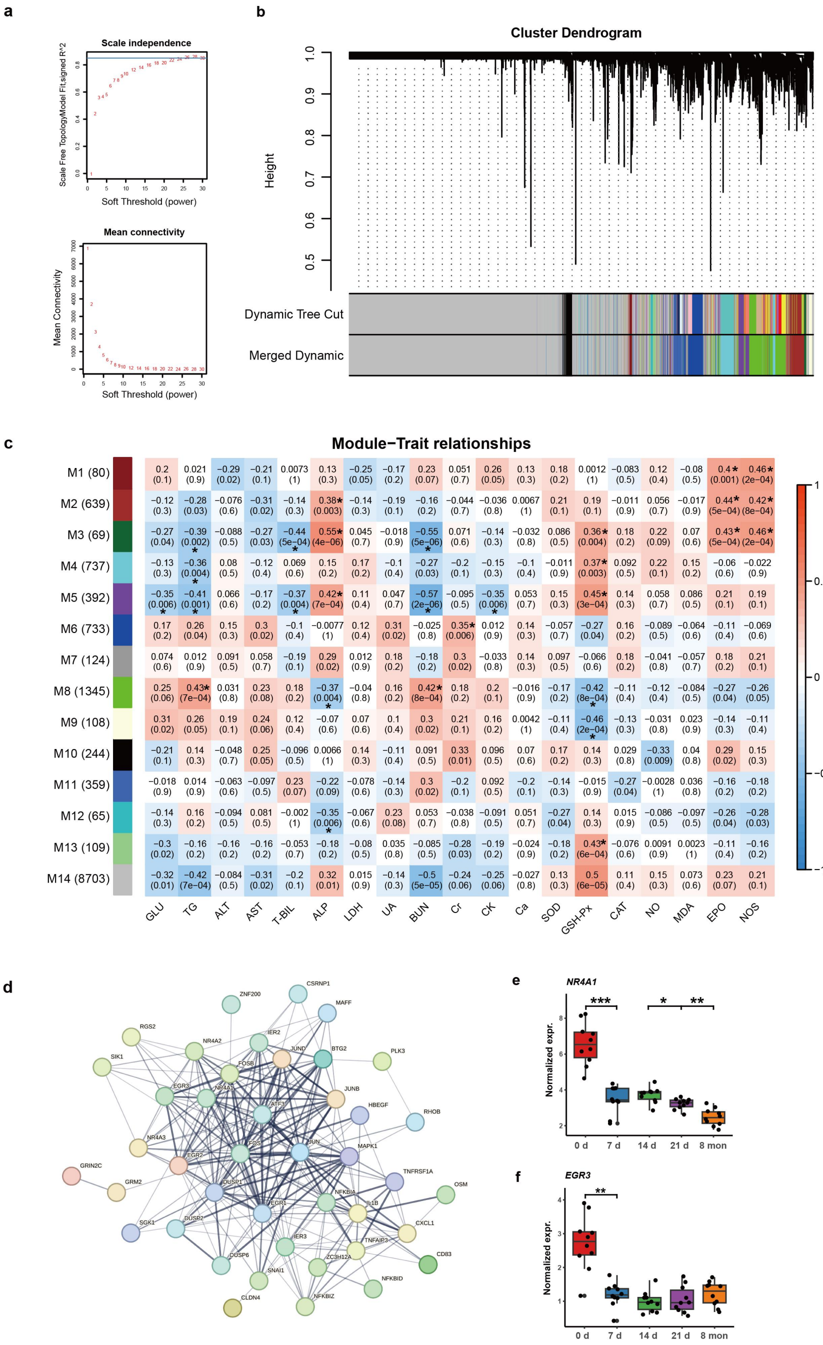

Supplementary Fig. 5 Weighted correlation network analysis (WGCNA). (a) Identification of optimal β-value. (b) The weighted gene co-expression network is constructed in blood. Colors represent gene co-expression modules. (c) Gene modules (M1-M14) associated with 19 blood bio-indicators (GLU, glucose; TG, triglycerides; ALT, alanine transaminase; AST, aspartate aminotransferase; T-BIL, total bilirubin; ALP, alkaline phosphatase; LDH, lactate dehydrogenase; UA, uric acid; BUN, blood urea nitrogen; Cr, creatinine; CK, cardiac enzymes; Ca, calcium; SOD, superoxide dismutase; GSH-Px, glutathione peroxidase; CAT, catalase; NO, nitric oxide; MAD, malondialdehyde; EPO, erythropoietin and NOS, nitric oxide synthase). The statistical

significance of module-trait relationship is corrected for multiple testing using the BH method, * means FDR < 0.05. The values in the brackets are the numbers of genes in corresponding modules. (d) Protein-protein interaction network analysis (STRING database v11) for gene module 3. (e-f) Gene examples of gene module 3. The expression changes of *NR4A1* (top) and *EGR3* (bottom) with time. Boxplots are represented by minima, 25% quantile, median, 75% quantile, and maxima with data points. P values from two-sided Wilcoxon rank sum test, * $P < 0.05$, ** $P < 0.01$, *** $P < 0.001$.

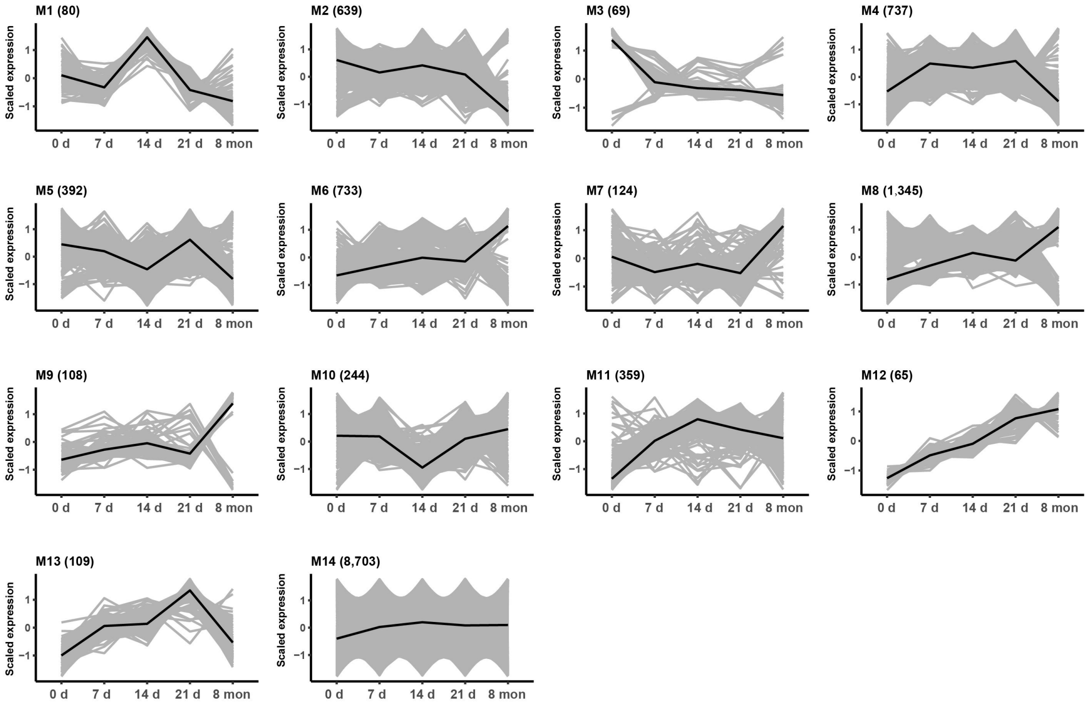

Supplementary Fig. 6 Time-series expression of 14 gene modules from WGCNA. The expression changes of 14 gene modules (M1-M14) with time.

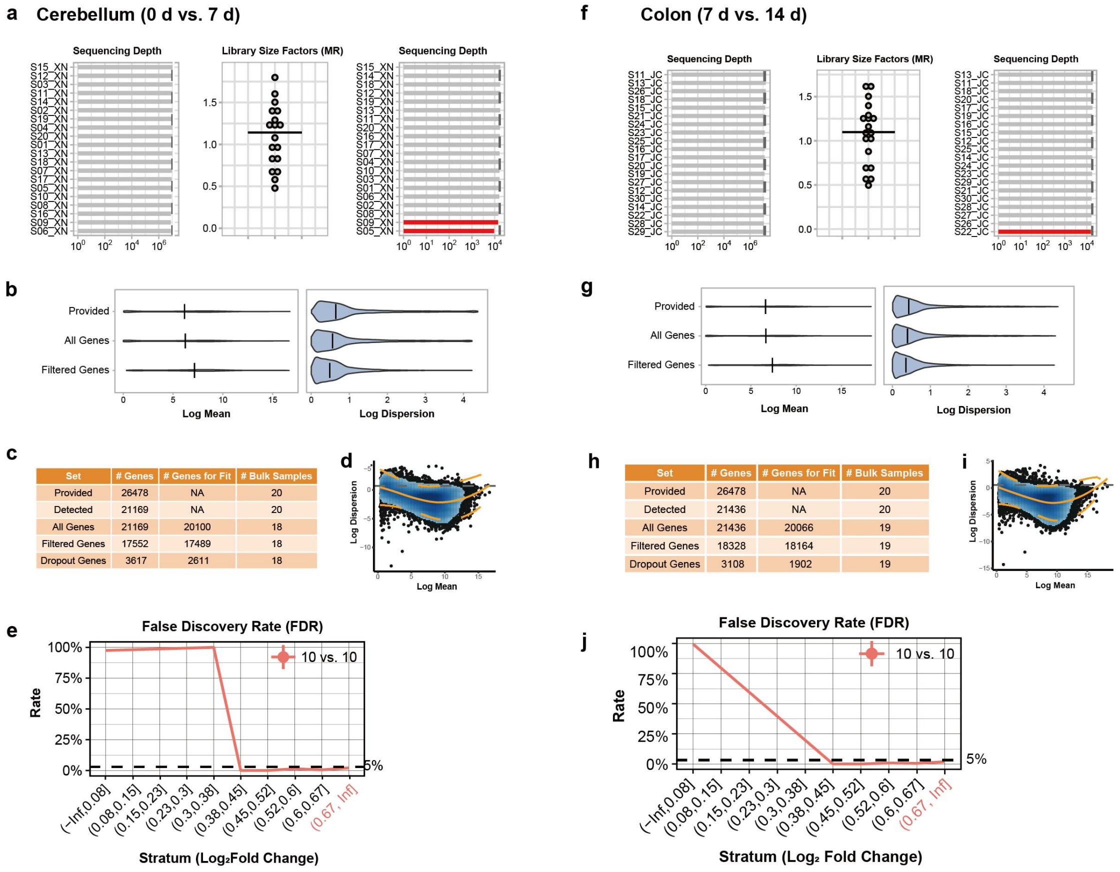

Supplementary Fig. 7 Power analysis for DEGs between adjacent time points. (a-e) Power analysis for samples from cerebellum in the comparison of "0 d vs. 7 d". (a) Quality control metrics for sequencing depth (left), library size factors with median value (black line) for the filtered data set (middle) and sequencing depth for detected genes (right). MR, normalization of median ratio method. (b) Marginal distribution of gene mean and dispersion per estimation set. (c) Number of genes and samples per estimation set. (d) Local polynomial regression fit between mean and dispersion estimates with variability band per gene (yellow). (e) Conditional false discovery rate (FDR) under the actual sample size per stratum. The stratum of the threshold used in the study is marked in pink. (f-j) Similar to a-e, power analysis for samples from the colon in the comparison of "7 d vs. 14 d".

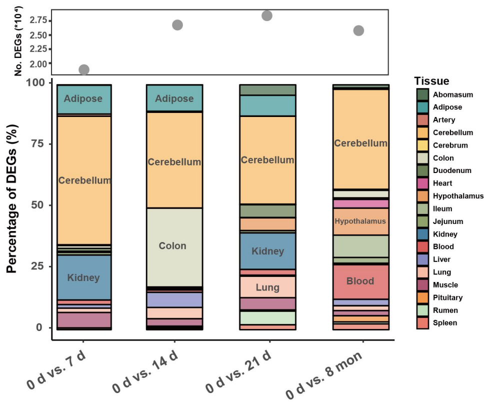

Supplementary Fig. 8 Numbers of differentially expressed genes (DEGs) (top) and percentages of DEGs (bottom) between low altitude Hu sheep (0 d) and high altitude Hu sheep (7 d, 14 d, 21 d and 8 mon) across tissues. Source Data are provided as Source Data file.

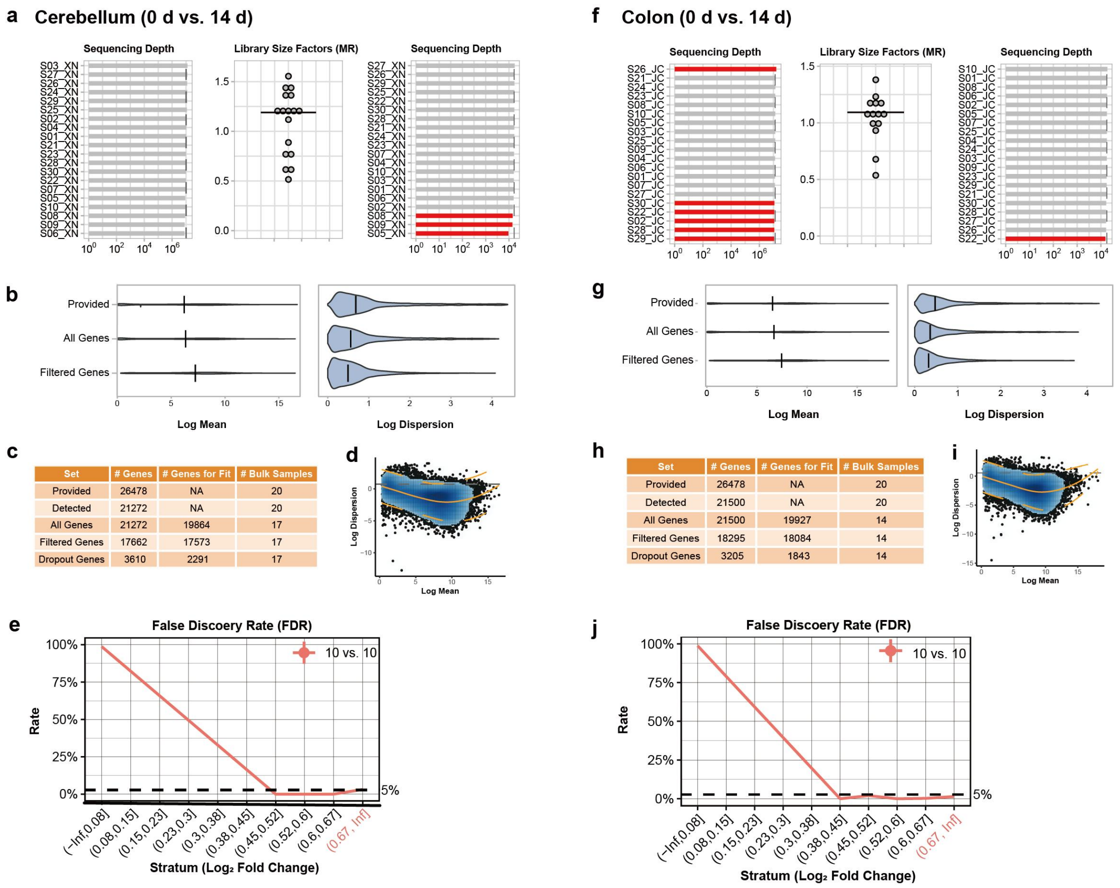

Supplementary Fig. 9 Power analysis for DEGs in cerebellum and colon between low altitude Hu sheep (0 d) and high altitude Hu sheep (14 d). (a-e) Power analysis for samples from cerebellum in the comparison of "0 d vs. 14 d". (a) Quality control metrics for sequencing depth (left), library size factors with median value (black line) for the filtered data set (middle) and sequencing depth for detected genes (right). MR, normalization of median ratio method. (b) Marginal distribution of gene mean and dispersion per estimation set. (c) Number of genes and samples per estimation set. (d) Local polynomial regression fit between mean and dispersion estimates with variability band per gene (yellow). (e) Conditional false discovery rate (FDR) under the actual sample size per stratum. The stratum of the threshold used in the study is marked in pink. (f-j) Similar to a-e, power analysis for samples from the colon in the comparison of "0 d vs. 14 d".

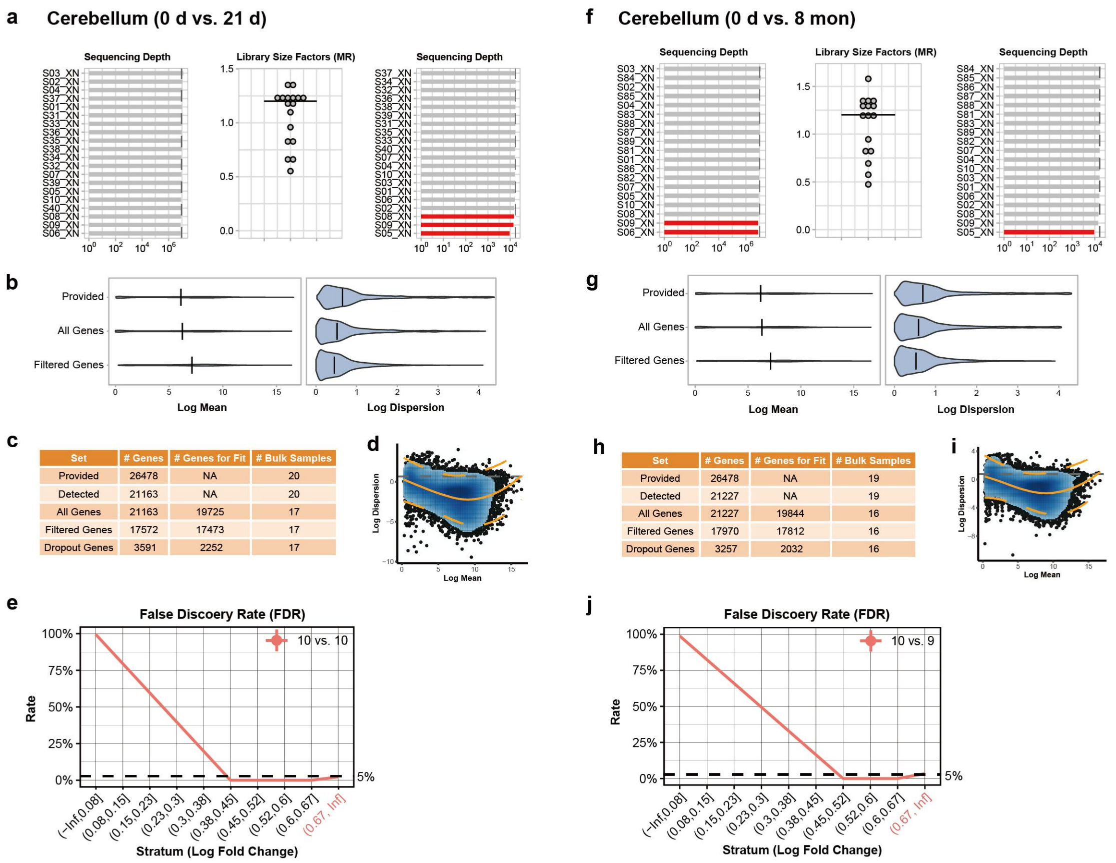

Set	# Genes	# Genes for Fit	# Bulk Samples
Provided	26478	NA	20
Detected	21163	NA	20
All Genes	21163	19725	17
Filtered Genes	17572	17473	17
Dropout Genes	3591	2252	17

Set	# Genes	# Genes for Fit	# Bulk Samples
Provided	26478	NA	19
Detected	21227	NA	19
All Genes	21227	19844	16
Filtered Genes	17970	17812	16
Dropout Genes	3257	2032	16

Supplementary Fig. 10 Power analysis for DEGs in cerebellum between low altitude Hu sheep (0 d) and high altitude Hu sheep (21 d and 8 mon). (a-e) Power analysis for samples from cerebellum in the comparison of “0 d vs. 21 d”. (a) Quality control metrics for sequencing depth (left), library size factors with median value (black line) for the filtered data set (middle) and sequencing depth for detected genes (right). MR, normalization of median ratio method. (b) Marginal distribution of gene mean and dispersion per estimation set. (c) Number of genes and samples per estimation set. (d) Local polynomial regression fit between mean and dispersion estimates with variability band per gene (yellow). (e) Conditional false discovery rate (FDR) under the actual sample size per stratum. The stratum of the threshold used in the study is marked in pink. (f-j) Similar to a-e, power analysis for samples from the cerebellum in the comparison of “0 d vs. 8 mon”

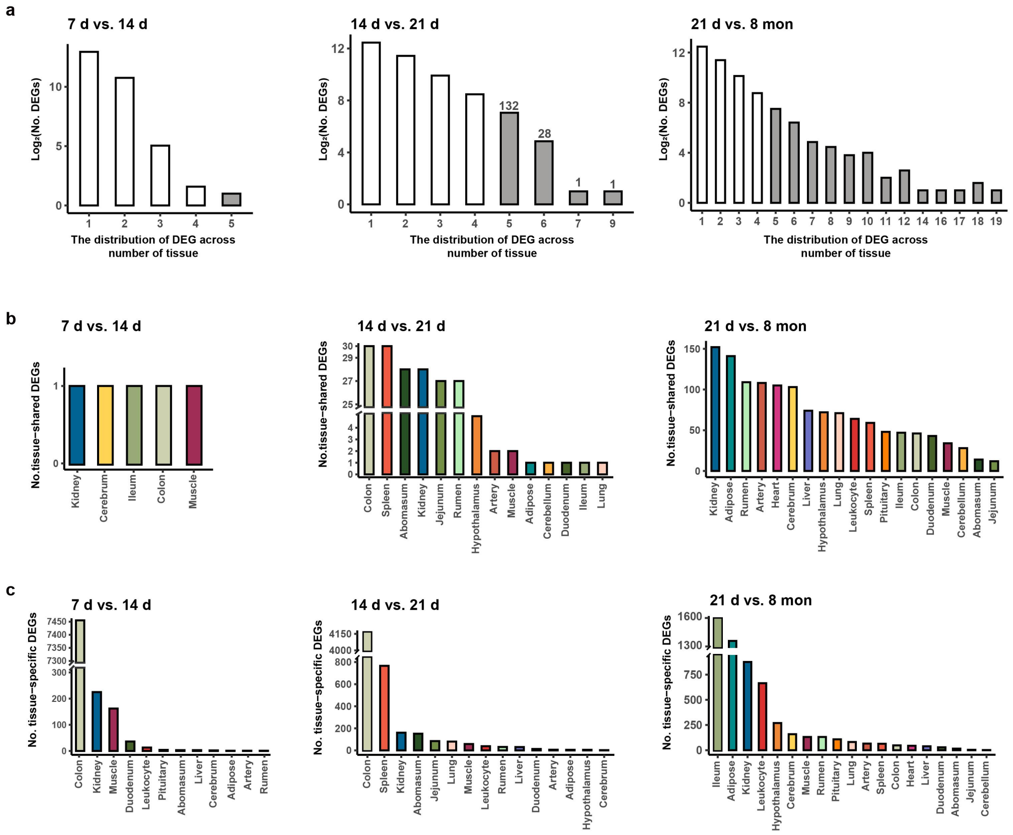

Supplementary Fig. 11 The distribution of tissue-shared and tissue-specific DEGs. (a) The distribution of DEGs across the number of tissues between different comparisons. (b) The number of tissue-shared DEGs across tissues between different comparisons. (c) The number of tissue-specific DEGs across tissues between different comparisons. Source Data are provided as Source Data file.

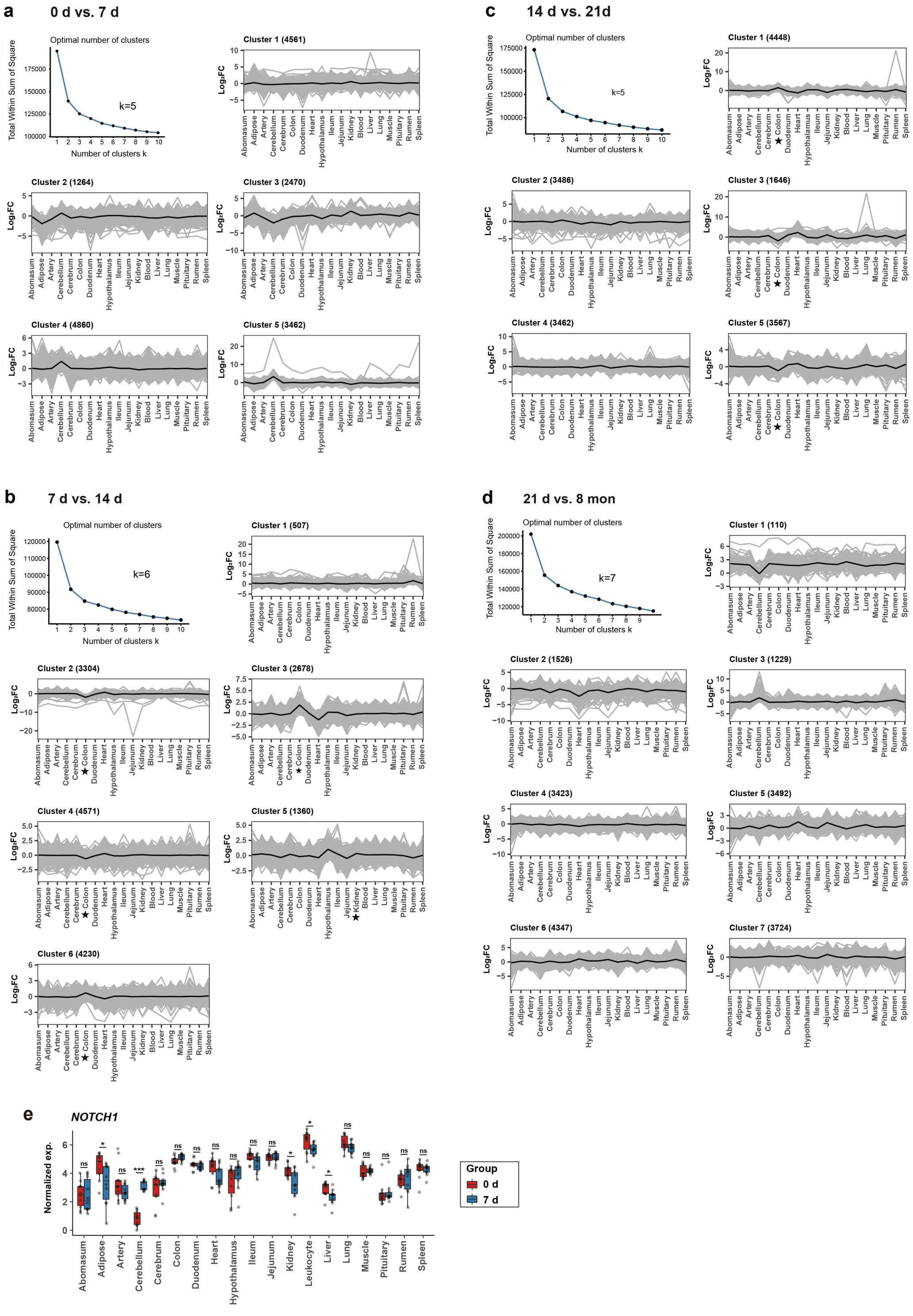

Supplementary Fig. 12 Multi-tissue interactions in different adjacent time point comparisons. (a-d) Multi-tissue interactions in “0 d vs. 7 d” (a), “7 d vs. 14 d” (b), “14 d vs. 21 d” (c) and “21 d vs. 8 mon” (d) comparisons. The average values of log_2FC for each cluster are denoted with black lines. (e) The expression level of *NOTCH1* in 0 d and 7 d across tissues. Boxplots are represented by minima, 25% quantile, median, 75% quantile, and maxima with data points. *P*-values from

two-sided Wilcoxon rank sum test, * $P < 0.05$, ** $P < 0.01$, *** $P < 0.001$. Source Data are provided as Source Data file.

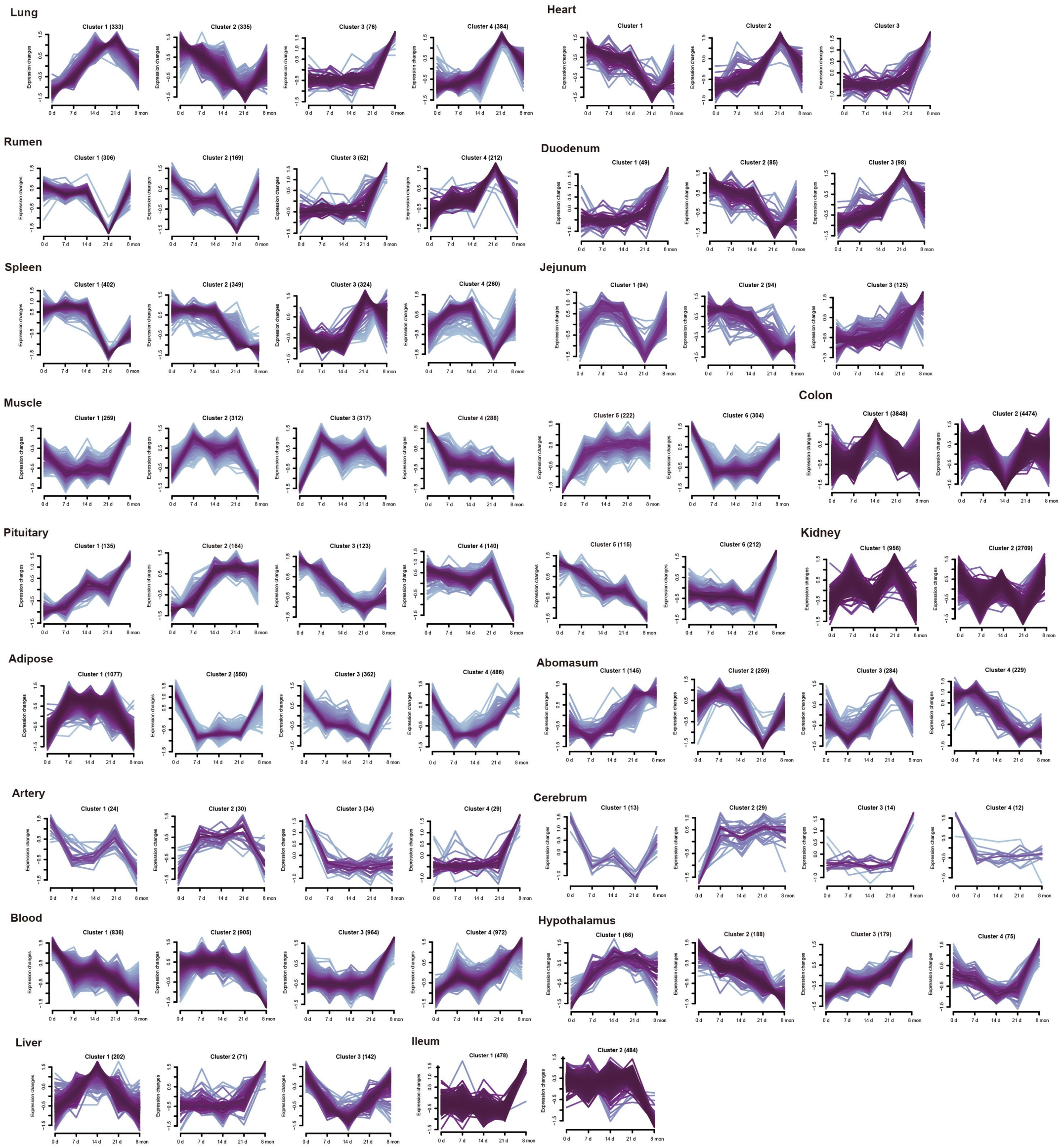

Supplementary Fig. 13 Fuzzy *c*-means clustering identified gene expression patterns of DCGs across tissues. Source Data are provided as Source Data file.

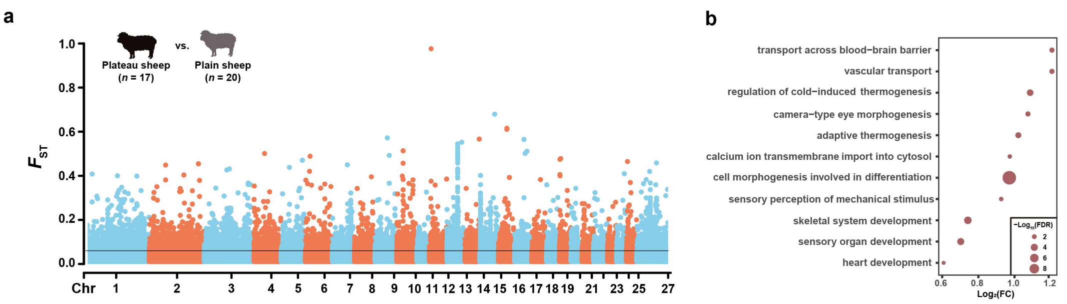

Supplementary Fig. 14 Selective sweep analysis. (a) Genome-wide distribution of pairwise F_{ST} values between between low altitude (n = 20) and high altitude sheep (n = 17). The top 5% significant threshold values of pairwise F_{ST} (F_{ST} = 0.0559) is denoted by black line. Chr, chromsome. (b) GO enichment of candidate selective genes identified from a. FDR < 0.05 is set as threshold. Source Data are provided as Source Data file.

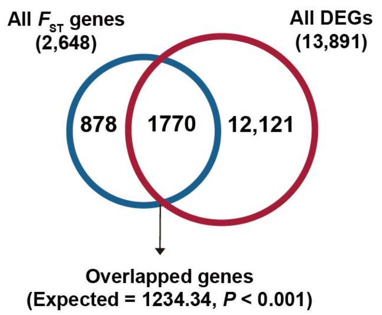

Supplementary Fig. 15 Permutation test for overlapped genes. (a) Permutation test for the overlapped genes between all F_{ST} genes and all differentially expressed genes (DEGs) within Hu sheep across tissues. The two-sided permutation test was performed with 1,000 times shuffle.

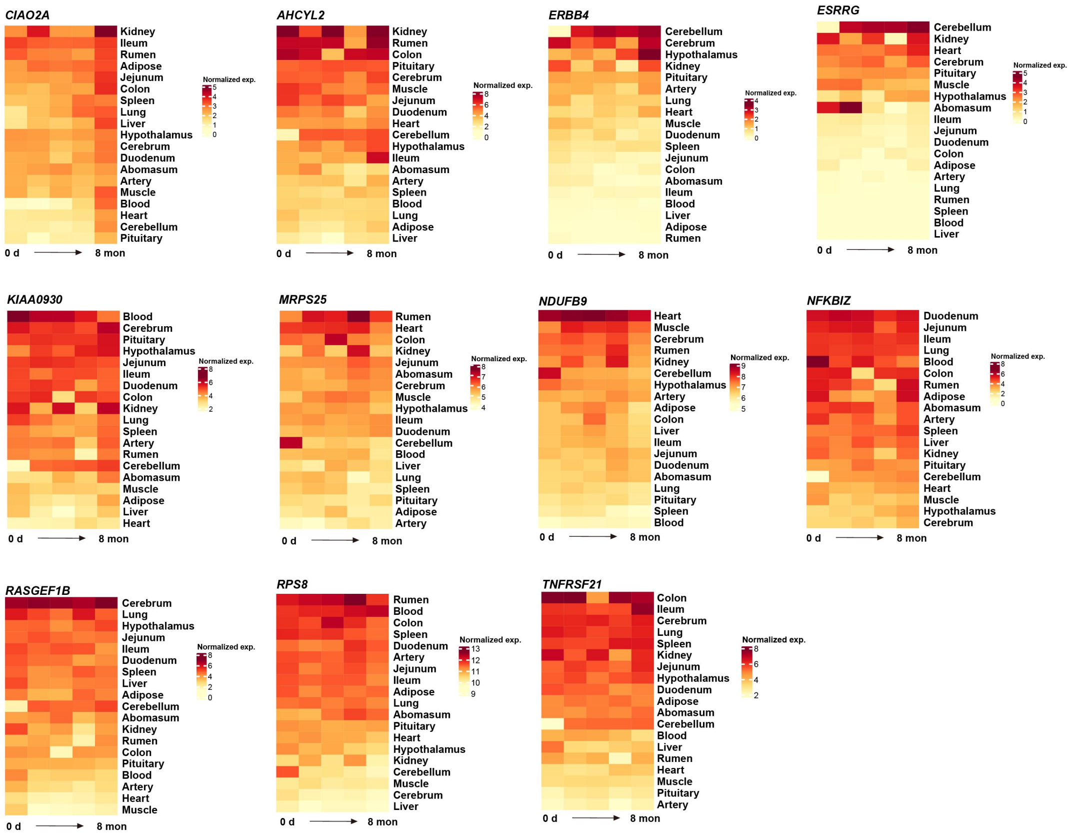

Supplementary Fig. 16 Expression of common tissue-shared F_{ST} genes. The expression changes of common tissue-shared F_{ST} genes with time across tissues.

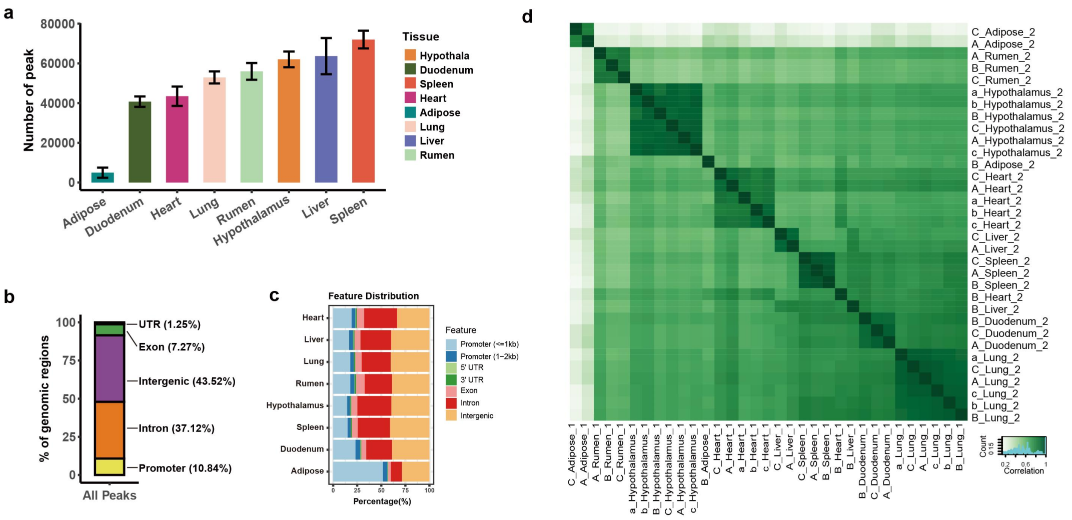

Supplementary Fig. 17 Data summary of ATAC-Seq. (a) The number of peaks across tissues. Error bars represent standard error, $n = 2$ independent replicates. (b) The overall distribution of peaks in genomic regions. (c) The distribution of peaks in genomic regions across tissues. (d) Pearson's correlation between all ATAC-Seq samples based on average peak density. Source Data are provided as Source Data file.

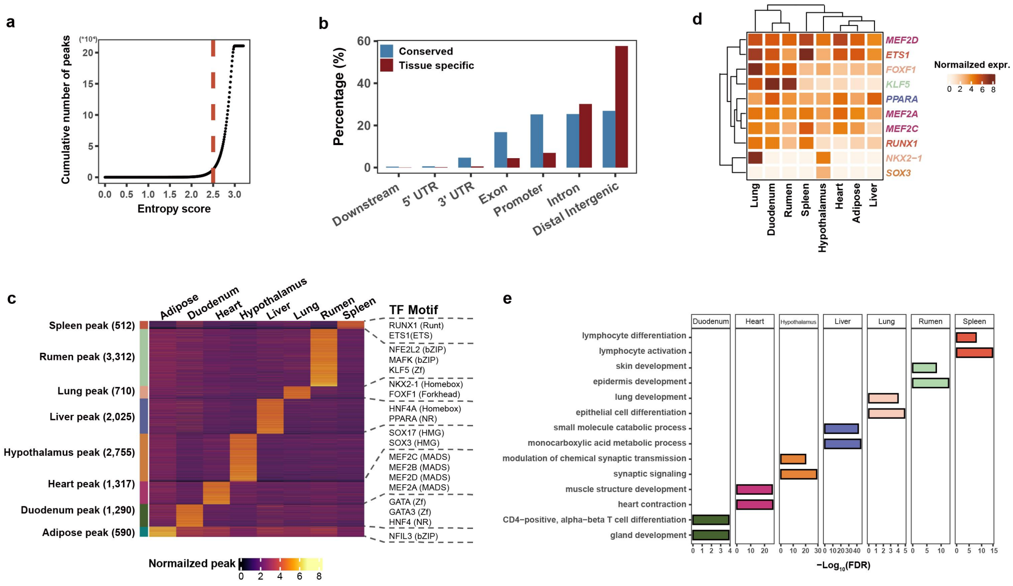

Supplementary Fig. 18 Tissue-specific peaks and motifs. (a) The identification of tissue-specific peaks based on Shannon entropy score. Entropy score = 2.5 is set as threshold and marked with a red line. (b) The distribution of conserved and tissue-specific peaks in genomic regions. (c) Heatmap shows the signal density of tissue-specific peaks, along with the representative transcription factor (TF) motif for each tissue. *P*-values come from the hypergeometric test. (d) Heatmap shows expression of TF target gene across tissues. The color code for tissues is the same as in c. (e) GO enrichment of tissue-specific peak linked genes across tissue. Two GO terms are displayed for each tissue. Source Data are provided as Source Data file.

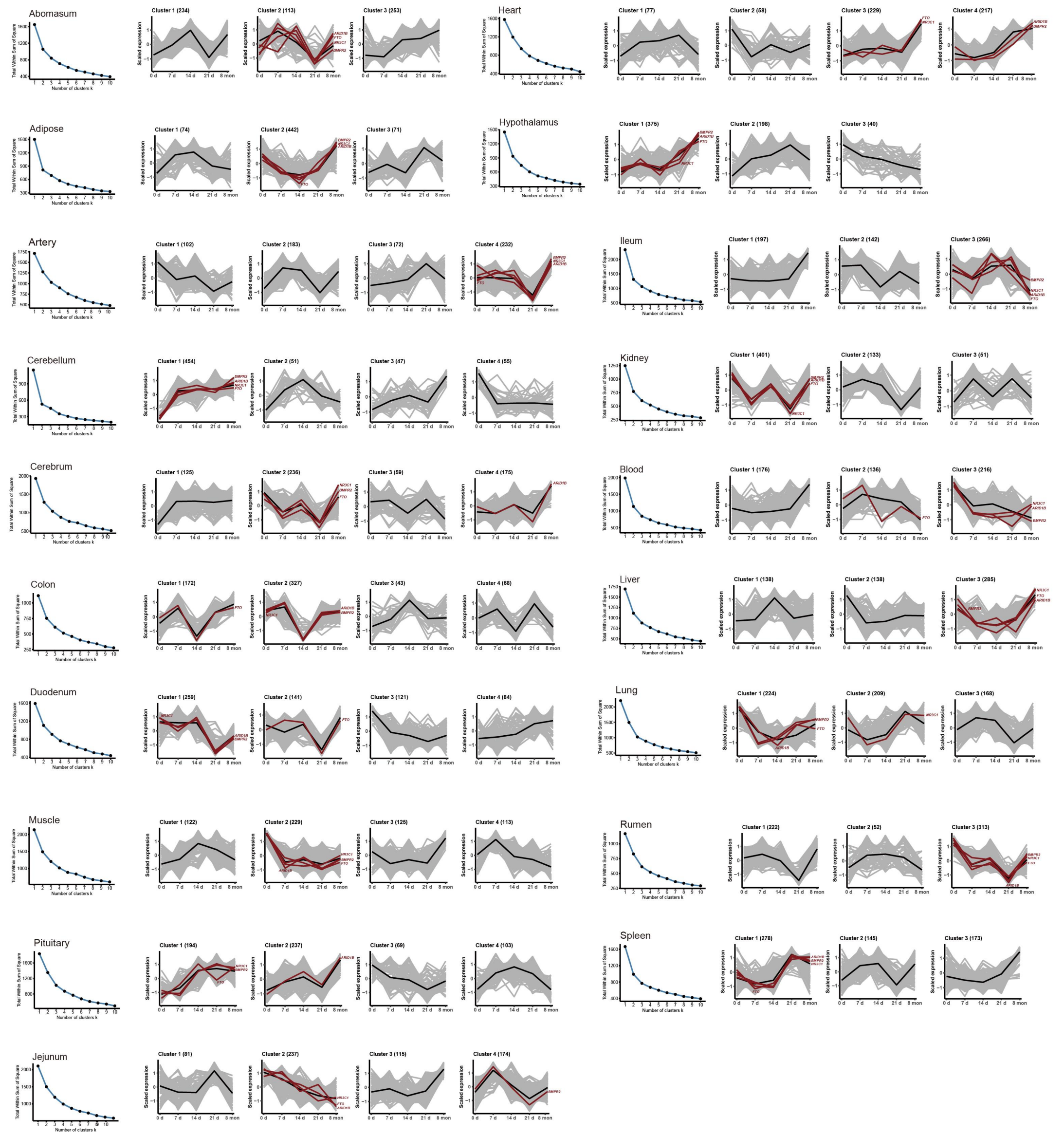

Supplementary Fig. 19 Time-series expression for mountain sickness candidate genes of human across tissues. We collected candidate genes (n = 613) associated with mountain sickness of human. The key candidate genes of pulmonary hypertension (*BMPR2*), polycythemia (*ARID1B*), pulmonary edema (*NR3C1*) and heart failure (*FTO*) are marked with red lines. The average value of expression for each cluster are denoted with black lines. Source Data are provided as Source Data file.

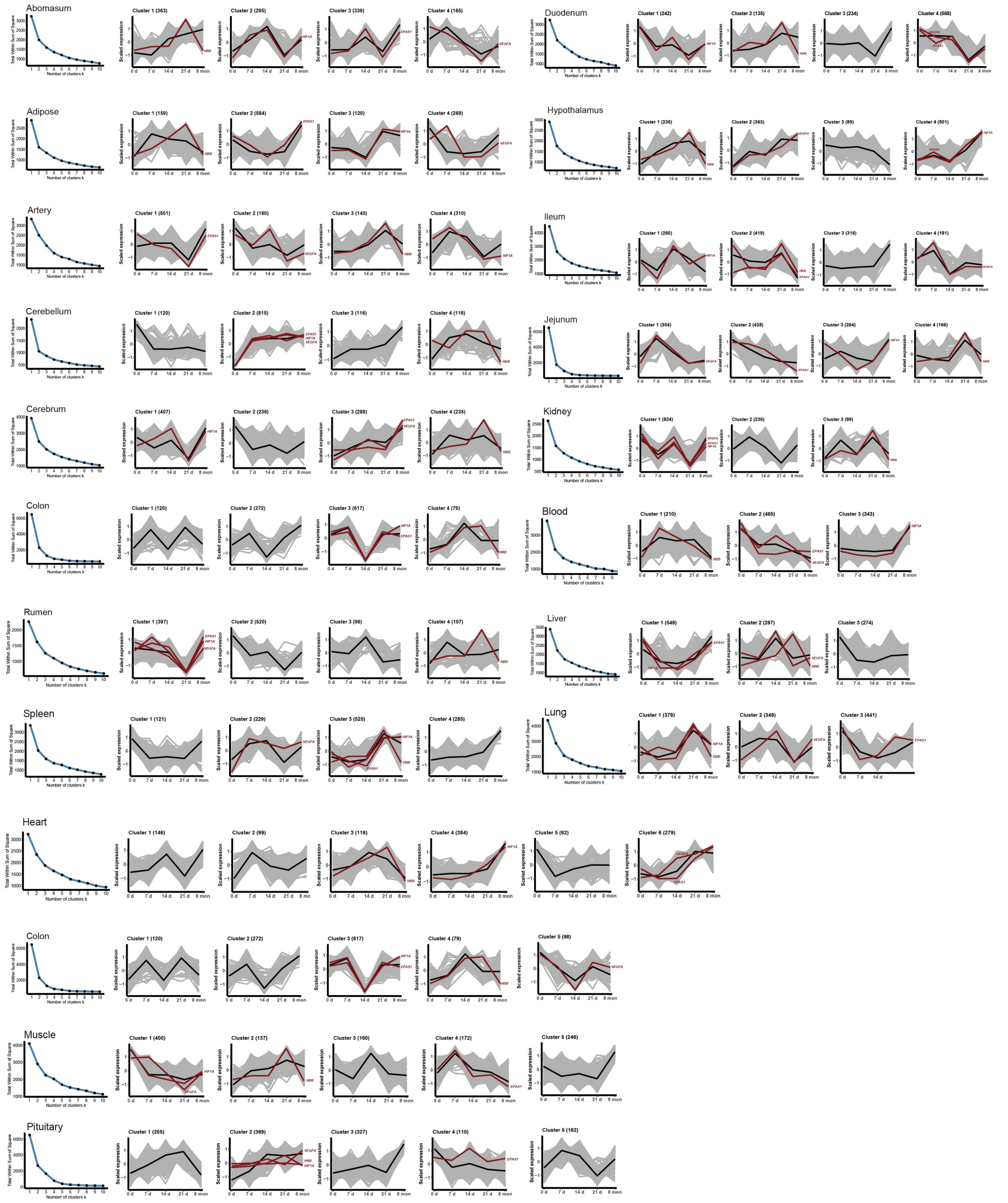

Supplementary Fig. 20 Time-series expression for high-altitude adaptation candidate genes of human across tissues. We collected candidate genes (n =1,207) associated with high-altitude adaptation of human. The key high-altitude adaptation candidate genes *HIF1A*, *EPAS1, VEGFA* and *HBB* are marked with red lines. The average value of expression for each cluster are denoted with black lines. Source Data are provided as Source Data file.

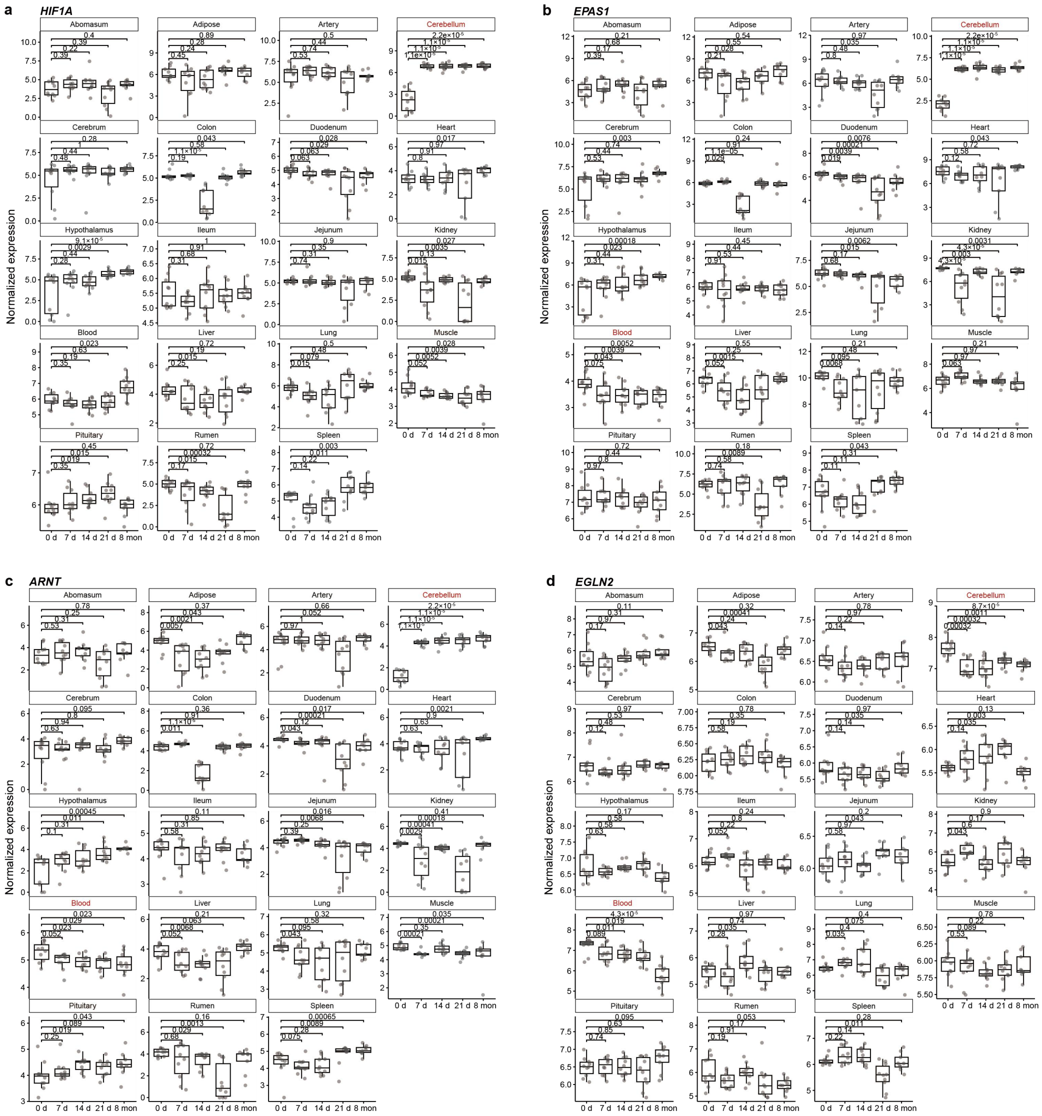

Supplementary Fig. 21 Time-series expression for HIF and PHD genes across tissues. (a-c) The expression changes of gene *HIF1A*, *EPAS1*, *ARN*T (i.e., HIF genes) with time across tissues. (d) Similar with a-c, but for *EGLN2* (i.e., PHD gene). Boxplots are represented by minima, 25% quantile, median, 75% quantile, and maxima with data points. Tissues with similar trends of expression change across four acclimatization time points are denoted in red. *P* values from the two-sided Wilcoxon rank sum test are marked.

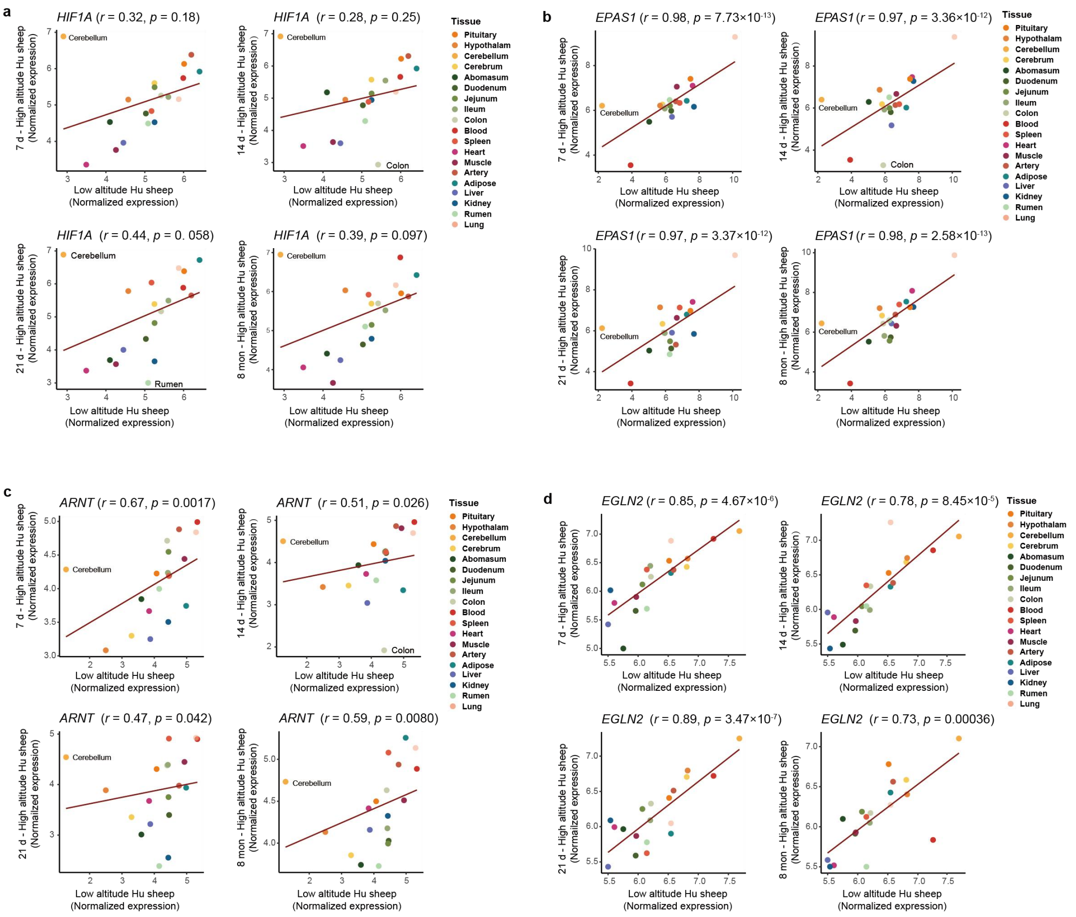

Supplementary Fig. 22 Pearson's correlation between the gene expression of low altitude Hu sheep (0 d) and high altitude Hu sheep (7 d, 14 d, 21 d and 8mon) across tissues for HIF genes (*HIF1A*, *EPAS1*, *ARNT*) and PHD gene (*EGLN2*). Tissues with the maximum extent of expression change across the four acclimatization time points when low altitude Hu sheep were translocated to high altitude are denoted. The two-sided *P* values are calculated by the linear regression model. (a) Pearson's correlation between the gene expression of low altitude Hu sheep (0 d) and high altitude Hu sheep (7 d, 14 d, 21 d and 8mon) across tissues for *HIF1A*. (b-d) Similar to (a), but for *EPAS1* (b), *ARNT* (c) and *EGLN2* (d) separately.

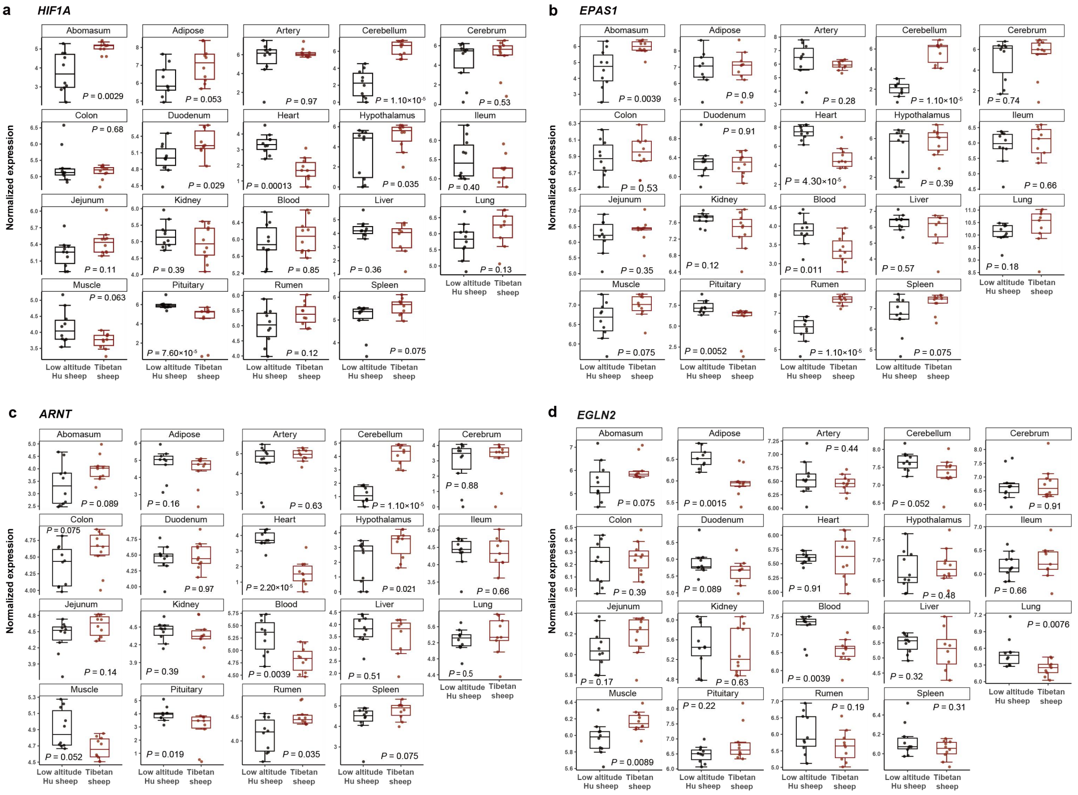

Supplementary Fig. 23 Gene expression comparison between low altitude Hu sheep and Tibetan sheep across tissues for HIF (*HIF1A*, *EPAS1*, *ARNT*) and PHD (*EGLN2*) genes. Boxplots are represented by minima, 25% quantile, median, 75% quantile, and maxima with data points. *P* values from the two-sided Wilcoxon rank sum test are denoted. (a) Gene expression comparison between low altitude Hu sheep and Tibetan sheep across tissues for *HIF1A*. (b-d) Similar to (a), but for *EPAS1* (b), *ARNT* (c) and *EGLN2* (d), respectively.

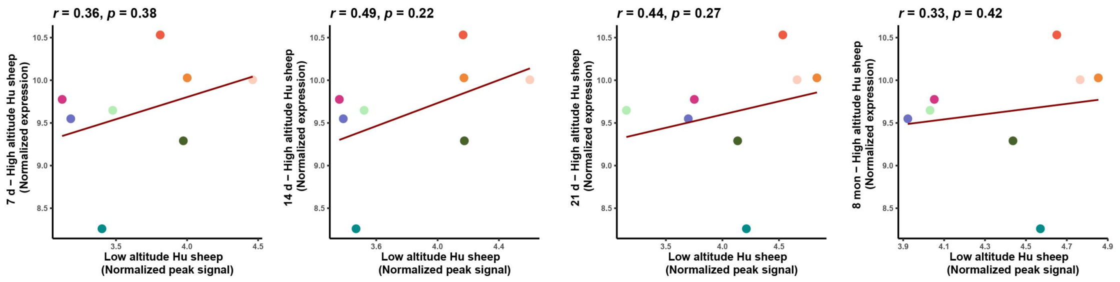

Supplementary Fig. 24 Linear regression between the peak signal value of low altitude Hu sheep and the expression of DEGs (between low altitude Hu sheep and high altitude Hu sheep at four acclimatization time points) in high altitude Hu sheep across tissues. Median values of peak signal and gene expression level were used for linear regression. *P* values were evaluated by Pearson correlation.

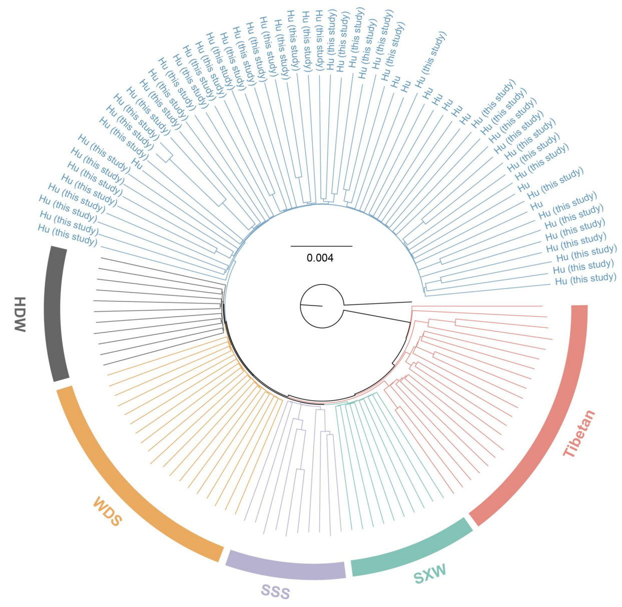

Supplementary Fig. 25 Neighbor-joining (NJ) tree of Tibetan sheep, Wadi sheep (WDS), small tailed Han sheep (SXW), large tailed Han sheep (HDW), Sishui fur sheep (SSS) and Hu sheep (Hu).

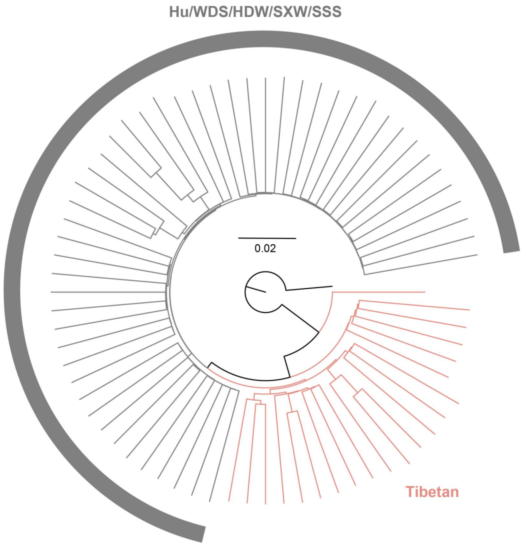

Supplementary Fig. 26 Neighbor-joining (NJ) tree of Tibetan sheep (pink) and five Chinese native sheep breeds (grey). Wadi sheep (WDS), small tailed Han sheep (SXW), large tailed Han sheep (HDW), Sishui fur sheep (SSS) and Hu sheep (Hu).

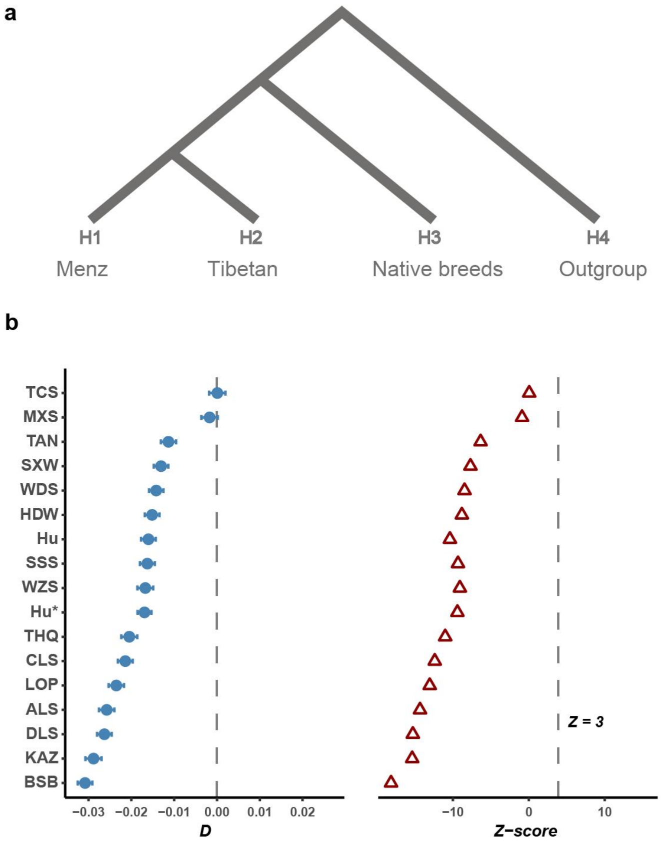

Supplementary Fig. 27 ABBA-BABA statistics between Tibetan sheep and 16 Chinese native sheep breeds. (a) Four-taxon model of ABBA-BABA statistics (i.e., *D* statistics), and H1-H4 represented reference, target, donor and outgroup populations, respectively. (b) Values of *D* statistics between Tibetan sheep and 16 Chinese native sheep breeds. Error bars represent *D* value ± SE. Samples in this study was marked with asterisk. TCS, Tengchong sheep; MXS, Minxian black fur sheep; TAN, Tan sheep; SXW, small tailed Han sheep; WDS, Wadi sheep; HDW, large tailed Han sheep; Hu, Hu sheep; SSS, Sishui fur sheep; WZS, Ujimqin sheep; THQ, Taihang fur sheep; CLS, Cele black sheep; LOP, Lop sheep; ALS, Altay sheep; DLS, Duolang sheep; KAZ, Kazakh sheep; BSB, Bashibai sheep. Statistical significances were shown by Z-scores. Source Data are provided as Source Data file.

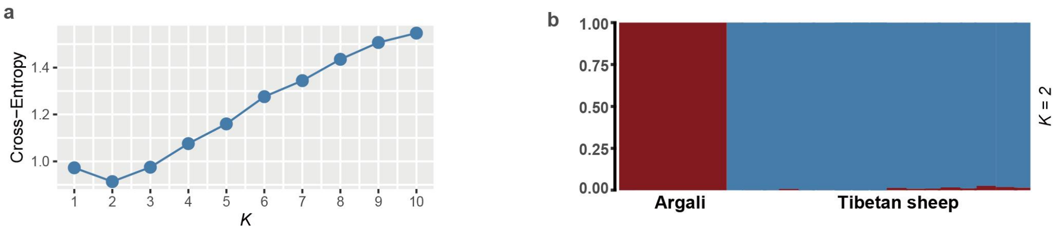

Supplementary Fig. 28 Population genetic structure of argali and Tibetan sheep. (a) Determination of optimal *K* value (*K* = 2). (b) Population genetic structure of argali and Tibetan sheep inferred using sNMF software. Source Data are provided as Source Data file.

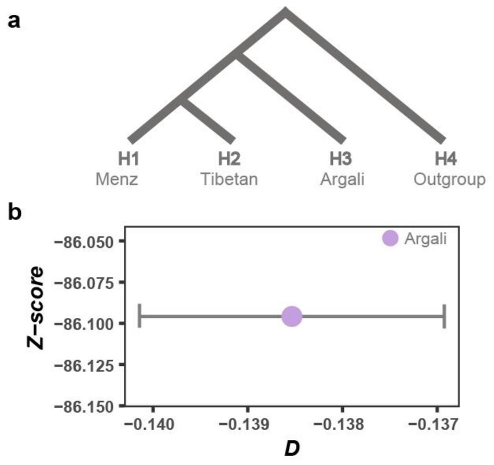

Supplementary Fig. 29 ABBA-BABA statistics between Tibetan sheep and wild sheep species (Argali). (a) Four-taxon model of ABBA-BABA statistics (i.e., *D* statistics), and H1-H4 represented reference, target, donor and outgroup populations, respectively. (b) Values of *D* statistics between Tibetan sheep and argali. Error bars represent *D* value ± SE. Source Data are provided as Source Data file.

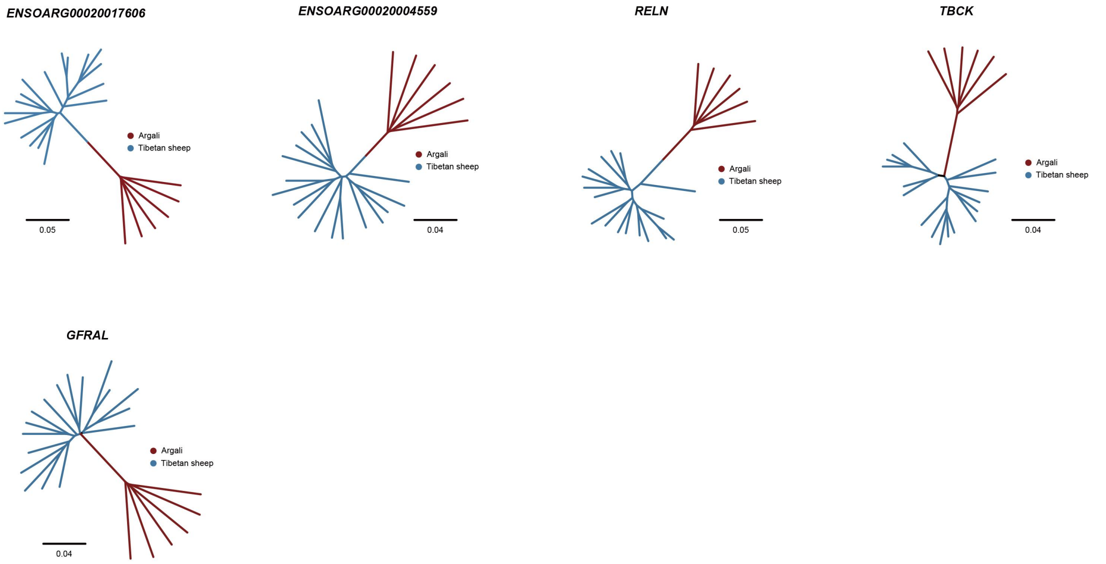

Supplementary Fig. 30 Gene tree of the top 5 F_{ST} genes (low altitude sheep vs. Tibetan sheep) for Tibetan sheep and argali based on the SNPs located in the 50 kb up and downstream of corresponding F_{ST} genes.
