# Supplementary material for: A time-resolved multi-omics atlas of transcriptional regulation in response to high-altitude hypoxia across whole-body tissues
Source: Nat Commun. 2024 May 10;15:3970. doi: 10.1038/s41467-024-48261-w (PMC11087590; doi:10.1038/s41467-024-48261-w)
Supplement: Supplementary file 3 — Description of additional supplementary files [file 41467_2024_48261_MOESM3_ESM.pdf]

Description of Additional Supplementary Files

Supplementary Data 1-40:

File Name: Supplementary Data 1
Description: Summary of tissue collection.

File Name: Supplementary Data 2
Description: Detailed information of sequencing samples in this study.

File Name: Supplementary Data 3
Description: Data summary of 49 WGS data.

File Name: Supplementary Data 4
Description: Data summary of 1,277 RNA-Seq data.

File Name: Supplementary Data 5
Description: Data summary of 66 ATAC-Seq data.

File Name: Supplementary Data 6
Description: Data summary of six scRNA-Seq datasets.

File Name: Supplementary Data 7
Description: Details of blood parameters.

File Name: Supplementary Data 8
Description: The value of 20 blood parameters.

File Name: Supplementary Data 9
Description: Detailed information of public WGS and Hi-C data.

File Name: Supplementary Data 10
Description: Data summary of WGS and Hi-C data.

File Name: Supplementary Data 11
Description: Statistics for 20 parameters.

File Name: Supplementary Data 12
Description: GO term enrichments of tissue-specific genes. *P* values are calculated by one-sided hypergeometric test. Multiple correction is based on the Benjamini–Hochberg method.

File Name: Supplementary Data 13

Description: GO term enrichments for each gene module in WGCNA. *P* values are calculated by one-sided hypergeometric test. Multiple correction is based on Benjamini–Hochberg method.

File Name: Supplementary Data 14
Description: Differentially expressed genes (DEGs) between low altitude Hu sheep (0d) and translocated high altitude Hu sheep (7 d, 14 d, 21 d and 8 mon) across tissues. P values are calculated by two-sided Wald test. Multiple correction is based on the Benjamini–Hochberg method.

File Name: Supplementary Data 15
Description: The distribution of differentially expressed genes (DEGs) across tissue for each adjacent time point comparison.

File Name: Supplementary Data 16
Description: Detail of the distribution of DEGs across tissue for each adjacent time point comparison.

File Name: Supplementary Data 17
Description: GO term enrichments of tissue-shared genes and tissue-specific DEGs for each adjacent time point comparison. *P* values are calculated by one-sided hypergeometric test. Multiple correction is based on the Benjamini–Hochberg method.

File Name: Supplementary Data 18
Description: *c*-means clustering for dynamically changed genes (DCGs) across tissues.

File Name: Supplementary Data 19
Description: Top 5 % F_{ST} regions and corresponding genes.

File Name: Supplementary Data 20
Description: GO term enrichments for candidate selected (top 5% F_{ST}) genes. P values are calculated by one-sided hypergeometric test. Multiple correction is based on the Benjamini–Hochberg method.

File Name: Supplementary Data 21
Description: Differentially expressed genes between low altitude Hu sheep and Tibetan sheep in each of the tissues. *P* values are calculated by two-sided Wald test. Multiple correction is based on the Benjamini–Hochberg method.

File Name: Supplementary Data 22
Description: The distribution of FST gene in DCGs and in inter-breed (low altitude Hu sheep vs. Tibetan sheep) DEGs across tissues.

File Name: Supplementary Data 23

Description: Permutation test (n = 1,000) of overlaps across tissues. P value is calculated by two-sided permutation test.

File Name: Supplementary Data 24
Description: Common shared F_{ST} genes in DCGs and inter-breed DEGs across tissues.

File Name: Supplementary Data 25
Description: Human Phenome-wide association analysis (Phe-WAS) results of common shared F_{ST} genes. P values are calculated by two-sided Chi-squared test. Multiple correction is based on the Benjamini-Hochberg method.

File Name: Supplementary Data 26
Description: Tissue-specific motifs (HOMER software). P values are calculated by one-sided hypergeometric test. Multiple correction is based on the Benjamini-Hochberg method.

File Name: Supplementary Data 27
Description: Motif for differential accessible regions (DARs) detected from three ewe groups. P values are calculated by one-sided hypergeometric test. Multiple correction is based on the Benjamini-Hochberg method.

File Name: Supplementary Data 28
Description: GO term enrichments for tissue-specific and conserved peak target genes. P values are calculated by one-sided hypergeometric test. Multiple correction is based on the Benjamini-Hochberg method.

File Name: Supplementary Data 29
Description: Intersection of differential DAR-linked gene and DEG between same comparison groups.

File Name: Supplementary Data 30
Description: GO term enrichments for common regulated genes between groups across tissues. P values are calculated by one-sided hypergeometric test. Multiple correction is based on the Benjamini-Hochberg method.

File Name: Supplementary Data 31
Description: GO term enrichments for common regulated genes between lamb groups. P values are calculated by one-sided hypergeometric test. Multiple correction is based on the Benjamini-Hochberg method.

File Name: Supplementary Data 32
Description: High-altitude adaptation candidate genes of human collected from previous studies.[1]

File Name: Supplementary Data 33
Description: Mountain sickness candidate genes of human collected from previous studies.

File Name: Supplementary Data 34
Description: Pearson's correlation of expression between sheep and human in high-altitude adaptation and mountain sickness genes. The two-sided P values are calculated by the linear regression model.

File Name: Supplementary Data 35
Description: Result of k-means clustering for high-altitude adaptation genes and mountain sickness genes of human.

File Name: Supplementary Data 36
Description: Cell types identified from scRNA-Seq in lung.

File Name: Supplementary Data 37
Description: Marker genes in lung from scRNA-Seq data. P values are calculated by two-sided Wilcoxon rank sum test.

File Name: Supplementary Data 38
Description: F_{ST} distance between Tibetan sheep and five Chinese native sheep breed

File Name: Supplementary Data 39
Description: WGS datasets for phylogenetic relationship and introgression analyses.

File Name: Supplementary Data 40
Description: Statistics of public WGS datasets in phylogenetic relationship and introgression analyses.
