# Supplementary material for: A time-resolved multi-omics atlas of transcriptional regulation in response to high-altitude hypoxia across whole-body tissues
Source: Nat Commun. 2024 May 10;15:3970. doi: 10.1038/s41467-024-48261-w (PMC11087590; doi:10.1038/s41467-024-48261-w)
Supplement: Supplementary file 5 — Reporting Summary [file 41467_2024_48261_MOESM5_ESM.pdf]

nature portfolio

Corresponding author(s): Meng-hua Li

Last updated by author(s): Mar 12, 2024

Reporting Summary

Nature Portfolio wishes to improve the reproducibility of the work that we publish. This form provides structure for consistency and transparency in reporting. For further information on Nature Portfolio policies, see our Editorial Policies and the Editorial Policy Checklist.

Statistics

For all statistical analyses, confirm that the following items are present in the figure legend, table legend, main text, or Methods section.

n/a	Confirmed	
☐	☒	The exact sample size (*n*) for each experimental group/condition, given as a discrete number and unit of measurement
☐	☒	A statement on whether measurements were taken from distinct samples or whether the same sample was measured repeatedly
☐	☒	The statistical test(s) used AND whether they are one- or two-sided<br>*Only common tests should be described solely by name; describe more complex techniques in the Methods section.*
☒	☐	A description of all covariates tested
☐	☒	A description of any assumptions or corrections, such as tests of normality and adjustment for multiple comparisons
☐	☒	A full description of the statistical parameters including central tendency (e.g. means) or other basic estimates (e.g. regression coefficient) AND variation (e.g. standard deviation) or associated estimates of uncertainty (e.g. confidence intervals)
☐	☒	For null hypothesis testing, the test statistic (e.g. *F*, *t*, *r*) with confidence intervals, effect sizes, degrees of freedom and *P* value noted<br>*Give P values as exact values whenever suitable.*
☒	☐	For Bayesian analysis, information on the choice of priors and Markov chain Monte Carlo settings
☐	☒	For hierarchical and complex designs, identification of the appropriate level for tests and full reporting of outcomes
☐	☒	Estimates of effect sizes (e.g. Cohen's *d*, Pearson's *r*), indicating how they were calculated

Our web collection on statistics for biologists contains articles on many of the points above.

Software and code

Policy information about availability of computer code

Data collection

Trimmomatic (v.0.36), BWA (v0.7.8), GATK (v4.1.2.0), fastp (v0.20.1), STAR (v2.7.9a), featureCounts (v2.0.3), VCFtools (v0.1.17), Genrich (v0.6.1), SAMtools (v1.11), Picard (v1.27), deepTools(v3.5.0),Hic-Pro (v2.9.0) HiCPeaks (v0.3.2) and TADLib (v0.4.2); Rstne (v0.16) ; ComplexHeatmap (v2.8.0); limma (v3.38.3); WGCNA (v1.12.0); DESeq2 (v1.32.0); maSigPro (v1.64.8); mFuzz (v.2.23.0); ClusterProfiler (v4.0.5); ChIPseeker (v1.28.3); IGV (v2.9.4); HOMER (v4.8); DiffBind (v3.6.1); Seurat (v.4.2.0); GENIE3 (v1.6.0); SCENIC (v1.1.2.2); CellPhoneDB (v2.0). Cytoscape (v3.7.1); AdmixTools (v7.0.1); sNMF (v1.2); SplitsTree (v4.18.3); PLINK (v1.90); EIGENSOFT (v.6.0.1); sva (v.3.40); DoubletFinder (v.2.0.3); biomaRt (v2.52.0); LiftOver (v377); factoextra (v1.0.7); powsimR (v0.090); preprocessCore (v1.40.0); BEDTools (v2.30.0).

Phenotypic data analysis implement in RStudio (v4.2.0) with functions: "shapiro.test", "aov", "kruskal.test", "TukeyHSD", "pairwise.wilcox.test". Genome-wide pairwise FST values with Python script popgenWindows.py (https://github.com/simonhmartin/genomics_general).

Data analysis

WGS analysis: Trimmomatic (v.0.36), BWA (v0.7.8), GATK (v4.1.2.0), VCFtools (v0.1.17), AdmixTools (v7.0.1), sNMF (v1.2), SplitsTree(v4.18.3), PLINK (v1.90), EIGENSOFT (v.6.0.1), Python script popgenWindows.py (https://github.com/simonhmartin/genomics_general).

RNA-Seq analysis: fastp (v0.20.1), STAR (v2.7.9a), featureCounts (v2.0.3), Rstne (v0.16), ComplexHeatmap (v2.8.0), limma (v3.38.3), WGCNA (v1.12.0), DESeq2 (v1.32.0), maSigPro (v1.64.8), mFuzz (v.2.23.0), ClusterProfiler (v4.0.5), sva (v.3.40), biomaRt (v2.52.0), factoextra (v1.0.7), powsimR (v0.090), preprocessCore (v1.40.0), Seurat (v.4.2.0).

ATAC-Seq analysis: fastp (v0.20.1), BWA (v0.7.8), Picard (v1.27), Genrich (v0.6.1), SAMtools (v1.11), deepTools (v3.5.0), ChIPseeker (v1.28.3), IGV (v2.9.4), HOMER (v4.8), DiffBind (v3.6.1), BEDTools (v2.30.0), LiftOver (v377).

Hi-C analysis: Trimmomatic (v.0.36), Hic-Pro (v2.9.0) HiCPeaks (v0.3.2), TADLib (v0.4.2).

Single-cell RNA-Seq: Seurat (v.4.2.0), GENIE3 (v1.6.0), SCENIC (v1.1.2.2), CellPhoneDB (v2.0), Cytoscape (v3.7.1), DoubletFinder (v.2.0.3)

Phenotypic data analysis implement in RStudio (v4.2.0) with functions: "shapiro.test", "aov", "kruskal.test", "TukeyHSD", "pairwise.wilcox.test".

For manuscripts utilizing custom algorithms or software that are central to the research but not yet described in published literature, software must be made available to editors and reviewers. We strongly encourage code deposition in a community repository (e.g. GitHub). See the Nature Portfolio guidelines for submitting code & software for further information.

Data

Policy information about availability of data

All manuscripts must include a data availability statement. This statement should provide the following information, where applicable:

- Accession codes, unique identifiers, or web links for publicly available datasets
- A description of any restrictions on data availability
- For clinical datasets or third party data, please ensure that the statement adheres to our policy

The data generated in this study, including WGS, RNA-seq, ATAC-seq and single-cell RNA-Seq data were deposited at Sequence Read Archive (SRA) database in NCBI under accession number PRJNA1053506 (https://www.ncbi.nlm.nih.gov/sra/?term=PRJNA1053506) for WGS, PRJNA1000743 (https://www.ncbi.nlm.nih.gov/sra/?term=PRJNA1000743) and PRJNA1001016 (https://www.ncbi.nlm.nih.gov/sra/?term=PRJNA1001016) for RNA-Seq and PRJNA1001505 (https://www.ncbi.nlm.nih.gov/sra/?term=PRJNA1001505) for ATAC-Seq and scRNA-Seq data.

The links for public datasets are list below: Hi-C (SRR19426890); WGS (https://www.ncbi.nlm.nih.gov/bioproject/PRJNA624020, https://www.ncbi.nlm.nih.gov/bioproject/?term=PRJNA645671, https://www.ncbi.nlm.nih.gov/bioproject/?term=PRJNA160933); GTEx database v8 (https://gtexportal.org/home/datasets); GWAS ATLAS (https://atlas.ctglab.nl/); RcisTarget database (v1.4.0 (https://resources.aertslab.org/cistarget)

Research involving human participants, their data, or biological material

Policy information about studies with human participants or human data. See also policy information about sex, gender (identity/presentation), and sexual orientation and race, ethnicity and racism.

Reporting on sex and gender	N/A
Reporting on race, ethnicity, or other socially relevant groupings	N/A
Population characteristics	N/A
Recruitment	N/A
Ethics oversight	N/A

Note that full information on the approval of the study protocol must also be provided in the manuscript.

Field-specific reporting

Please select the one below that is the best fit for your research. If you are not sure, read the appropriate sections before making your selection.

☒ Life sciences ☐ Behavioural & social sciences ☐ Ecological, evolutionary & environmental sciences

For a reference copy of the document with all sections, see nature.com/documents/nr-reporting-summary-flat.pdf

Life sciences study design

All studies must disclose on these points even when the disclosure is negative.

Sample size	No sample size calculation was performed in advance. For RNA-Seq data, we used 10 biological replicates for each sheep ewe groups and 6 biological replicates for lamb groups. And we excluded technical artifacts based on power analysis. For ATAC-Seq data, we used independent two biological replicates for each group in different tissues with at least technical duplicates. These sizes have previously been shown to be sufficient power to obtain reliable gene expression and chromatin accessibility data and determine statistical differences of investigated parameters.
Data exclusions	No data was excluded.
Replication	For RNA-Seq data, we collected 10 biological replicates for 19 different tissues in sheep ewe groups (low altitude Hu sheep, translocated high altitude Hu sheep at four acclimatization time points and Tibetan sheep). In addition, we collected six biological replicates in 10 tissues for three lamb groups (low altitude Hu lamb, high altitude Hu lamb and Tibetan lamb). For ATAC-Seq data, we collected two biological replicates for eight tissues in sheep ewe groups (low altitude Hu sheep, translocated high altitude Hu sheep for 8 months acclimatization and Tibetan sheep) and three tissues in lamb groups (low altitude Hu lamb, high altitude Hu lamb and Tibetan lamb), respectively. For each phenotypic

	indicator, values were measured from each individual over three times and were all recorded. The replication number is indicated in the legend of corresponding figures where applicable.
Randomization	Randomization was not relevant to this study. We controlled potential sources of confounding by (1) animals were raised in standardized housing. (2) feeding conditions and collection method of samples was unified.
Blinding	Blinding was not used because all analyses in the main text were performed on the premise that the samples are known.

Reporting for specific materials, systems and methods

We require information from authors about some types of materials, experimental systems and methods used in many studies. Here, indicate whether each material, system or method listed is relevant to your study. If you are not sure if a list item applies to your research, read the appropriate section before selecting a response.

Materials & experimental systems

n/a	Involved in the study	
☒	☐	Antibodies
☒	☐	Eukaryotic cell lines
☒	☐	Palaeontology and archaeology
☐	☒	Animals and other organisms
☒	☐	Clinical data
☒	☐	Dual use research of concern
☒	☐	Plants

Methods

n/a	Involved in the study	
☒	☐	ChIP-seq
☒	☐	Flow cytometry
☒	☐	MRI-based neuroimaging

Animals and other research organisms

Policy information about studies involving animals; ARRIVE guidelines recommended for reporting animal research, and Sex and Gender in Research

Laboratory animals	A total of 78 adult sheep were included in low-to-high altitude translocation experiment, including 60 sheep ewes (10 sheep raised in low altitude plain, 40 Hu sheep ewes were translocated from low altitude to high altitude and 10 Tibetan sheep ewes raised in high altitude) and 18 lambs (six lambs of low altitude Hu sheep, six lamb of translocated Hu sheep ewes after 8 months acclimatization and six lambs of Tibetan sheep ewes). Sheep ewes (~1.5 years) and lambs (~ 2months) were healthy and in good body condition.
Wild animals	No wild animals are used in the study.
Reporting on sex	To explore high altitude acclimatization across generations, we only used mature ewes (~1.5 years old) and their lambs in the three scenarios of the translocation experiment. For lambs, male and female were equal for each group (three male lambs and three female lambs). The details were summarized in Supplementary Data 2.
Field-collected samples	10 low altitude sheep ewes and 6 lambs were housed at ~ 350 m.a.s.l. on the Wanghu Livestock Farm in Neijiang City, Sichuan Province, China. 40 adults ewes and 3 rams of Hu sheep born and raised in the aforementioned Wanghu Livestock Farm were translocated to the Tibetan Sheep Breeding Farm of Sichuan Province (Aba Tibetan and Qiang Autonomous Prefecture, Sichuan Province, China) and produced 10 lambs after approximately 8 months. 10 adult ewes and 6 lambs of Tibetan sheep were housed at ~ 3,500 m.a.s.l. in Tibetan Sheep Breeding Farm. The ewes and lambs were housed and fed similar hay and silage corn, as previously described. and had ad libitum access to water and mineral salt. The farm houses was under natural temperature photoperiod. Animals were slaughtered by carotid artery exsanguination. Following sacrifice, tissues were isolated and placed on an ice board and then snap frozen in liquid nitrogen.
Ethics oversight	All experimental protocols in this study were reviewed and approved by the Institutional Animal Care and Use Committee of China Agricultural University (CAU20160628-2) and the local animal research ethics committee. Animal care, maintenance, procedures, and experimentation were performed in strict accordance with the guidelines and regulations approved by the Welfare and Ethics Committee of the Chinese Association for Laboratory Animal Sciences.

Note that full information on the approval of the study protocol must also be provided in the manuscript.

Plants

Seed stocks

Report on the source of all seed stocks or other plant material used. If applicable, state the seed stock centre and catalogue number. If plant specimens were collected from the field, describe the collection location, date and sampling procedures.

Novel plant genotypes

Describe the methods by which all novel plant genotypes were produced. This includes those generated by transgenic approaches, gene editing, chemical/radiation-based mutagenesis and hybridization. For transgenic lines, describe the transformation method, the number of independent lines analyzed and the generation upon which experiments were performed. For gene-edited lines, describe the editor used, the endogenous sequence targeted for editing, the targeting guide RNA sequence (if applicable) and how the editor was applied.

Authentication

Describe any authentication procedures for each seed stock used or novel genotype generated. Describe any experiments used to assess the effect of a mutation and, where applicable, how potential secondary effects (e.g. second site T-DNA insertions, mosiacism, off-target gene editing) were examined.
